# BRAF^V600E^ mutational status assessment in cutaneous melanocytic neoplasms in a group of the Egyptian population

**DOI:** 10.1186/s12935-023-02858-1

**Published:** 2023-02-03

**Authors:** Nada M. Yakout, Dina M. Abdallah, Doaa A. Abdelmonsif, Hassan Mahmoud Kholosy, Iman M. Talaat, Omayma Elsakka

**Affiliations:** 1 Pathology Department, Faculty of Medicine, Alexandria University, Alexandria, Egypt; 2 Medical Biochemistry Department, Genomics Research Lab, Faculty of Medicine, Alexandria University, Alexandria, Egypt; 3 Department of Plastic Surgery, Faculty of Medicine, Alexandria University, Alexandria, Egypt; 4 Clinical Sciences Department, College of Medicine, University of Sharjah, Sharjah, United Arab Emirates

**Keywords:** Melanoma, BRAFV600E, Bleaching, Egyptian, Immunohistochemistry

## Abstract

**Background:**

Melanocytic neoplasms range from banal nevi to malignant melanomas. The genetic background has been extensively studied in the Caucasian population. *BRAF* mutations were reported among the early driver mutations in nevogenesis. Nevertheless, the pathogenesis in the Egyptian population has not been elucidated.

**Aim and Methods:**

The present study was carried out to assess the sensitivity and specificity of immunohistochemistry (IHC) using the RM-08 clone in reference to allele-specific real-time PCR (CAST-PCR) for the detection of the *BRAF *^*V600E*^ mutation in 50 formalin-fixed paraffin-embedded blocks of melanocytic neoplasms with prior bleaching using hydrogen peroxide in Tris-HCL and Bovine Serum Albumin respectively.

**Results:**

IHC staining was interpreted using staining reaction (positive versus negative) and staining pattern (negative and heterogeneous versus homogenous). Using the staining pattern, the specificity increased from 73.3 to 88.2%, the negative predictive value increased from 73.3 to 100%, the diagnostic accuracy increased from 71.4 to 90.48% and the overall accuracy increased from 69.9 to 77.3%. The sensitivity and positive predictive value remained unchanged. The K-agreement coefficient increased from 0.364 (fair agreement) to 0.741 (good agreement) and was statistically significant (p = 0.00). Next-generation sequencing was performed in 11 cases, 8 cases with IHC-positive and *BRAF* ^wild type^ in addition to 3 cases that failed PCR analysis and revealed no *BRAF *^*V600E*^. No statistically significant difference was found in the clinicopathological parameters between *BRAF* ^V600E^ and *BRAF *^*wild−type*^ melanomas.

**Conclusions:**

These findings suggest that IHC staining homogeneity may be more accurate in predicting *BRAF* ^V600E^ mutational status. However, IHC cannot replace molecular methods.

## Introduction

Melanocytic neoplasms range from benign banal nevi to malignant melanomas, which are considered among the most aggressive malignancies. The incidence differs among different geographical regions. This may be attributed to racial variation, as well as differences in sun exposure. In the USA, it is ranked as the fifth most common malignancy in males [1]. In Egypt, it accounts for only 0.24% of malignancies [2].

Melanoma formation depends on the interplay between environmental risk and host susceptibility. The UV load, the pattern of sun exposure (intermittent versus chronic), anatomic location and driver mutations provide a basis for the classification of cutaneous melanocytic neoplasia, specifically melanomas in the Caucasian population according to the 4th edition of the WHO classification of skin tumours [3, 4]. Nevertheless, in dark-skinned individuals, in the Middle East and North Africa (MENA) region, including the Egyptian population, the pathogenesis of nevi and melanoma may be unclear due to few published data [2, 5].

One of the essential genes implicated in nevi and melanoma development is the *BRAF* gene, a human proto-oncogene located on the long arm of chromosome 7 (7q34). It encodes BRAF protein, a member of the Raf kinase family of proteins playing a pivotal role in the MAPK/ERK signalling pathway. The BRAF protein is a 766–amino acid-long protein composed of three conserved domains: the two regulatory domains and the third domain, which encodes the kinase domain, in the C-terminus [6].

The constitutive oncogenic activation of the BRAF protein promotes a continuous, uncontrolled stimulation of cell proliferation via phosphorylation of MEK and ERK [7]. This process occurs due to a missense mutation of the BRAF gene. To date, more than 30 mutations of the* BRAF* gene associated with human cancers have been identified, the most common of which is the *BRAF*^V600E^ mutation, in which hydrophilic glutamic acid (E) substitutes hydrophobic valine (V) at codon 600. Less commonly encountered mutations are grouped as *BRAF*.^non−V600E^ mutations [8].

Molecular testing is necessary to identify the mutational status and can be performed using different techniques, including the FDA-approved Cobas test, sequencing, and real-time PCR. Immunohistochemistry using a monoclonal antibody VE1 clone has been proposed as a surrogate for RT–PCR molecular testing [8–10].

This study assessed BRAF mutational status using IHC for BRAF ^V600E^ and competitive allele-specific real-time PCR (CAST-PCR) in 50 formalin-fixed paraffin-embedded (FFPE) tissue blocks representative of melanocytic neoplasms. The results of IHC testing were compared with the CAST-PCR results to determine the diagnostic accuracy and clinical value of IHC testing for detecting the *BRAF* ^V600E^ mutation.

## Materials and methods

### Patients

The current study comprised 50 retrospective excisional or incisional biopsies of cutaneous melanocytic neoplasms (29 melanomas and 21 nevi cases) collected from the archives of the Pathology Laboratory, Faculty of Medicine, Alexandria University during the period from January 2017 to January 2020. Ethical approval was granted by the Faculty of Medicine Ethics Committee (IRB no. 00007555, FWA NO. 00015712). The cases selected had sufficient tissue material as assessed by light microscopic examination of H&E-stained, formalin-fixed, paraffin-embedded tissue sections.

Several methods have been used to detect *BRAF* mutation status. Molecular methods were solely used to evaluate *BRAF*^*V600E*^ mutational status. With the development of the BRAF^*V600E*^ monoclonal antibody, the possibility of using immunohistochemistry (IHC) to detect the *BRAF*^*V600E*^ mutation has been reported. IHC is cost-effective and available as a routine technique and rapidly yields results.

### *Immunohistochemical staining for BRAF*^*V600E*^*:*

Four-micrometre-thick sections were cut from paraffin blocks and placed on positively charged slides. Melanin bleaching for moderately and heavily pigmented cases (17 melanoma cases and 12 nevi) using 0.5% diluted hydrogen peroxide (H2O2) in Tris-HCl and PBS was performed as described by Chung et al. [11]. Antigen retrieval was performed using sodium citrate buffer (0.01 M Na-citrate monohydrate, pH 6.0) in a microwave oven for 15 min. IHC staining was performed using BRAF monoclonal antibody (Clone: RM8, ThermoFisher Scientific, USA, dilution 1:300 in phosphate-buffered saline (PBS) according to the manufacturer’s protocol using a horseradish polymer (hrp) kit (ThermoFisher Scientific) and diaminobenzidine (DAB) as a chromogen. Nonneoplastic prostate and neoplastic colon and tissue sections without primary antibody incubation were included in the run as positive and negative controls, respectively.

All cases were scored by three pathologists who were not informed of the clinical course and diagnosis following the criteria proposed by Capper et al. [10]. The staining pattern was interpreted as homogenous and heterogeneous according to the staining pattern of tumour cells as described by Yancovitz [12].

### *BRAF*^*V600E*^* molecular analysis:*

Eight sections from paraffin blocks of the 50 cases were cut at a thickness of 10 μm and a surface area of 250 mm^2^. Genomic DNA was extracted according to the manufacturer’s instructions of the QIAamp DNA FFPE Tissue Kit (Qiagen; http://www.qiagen.com). Eleven control tissue samples were obtained from non-neoplastic tissues (normal pancreatic and normal skin tissues). This material was used before PCR runs to optimize the curves for reference genes.

### Competitive allele-specific real-time PCR (CAST-PCR)

Quantitative PCR for the *BRAF*^V600E^ mutation was performed in all 50 cases using competitive allele-specific TaqMan mutation detection assays: Mutation Allele Assay (4465804, Hs00000111_mu) and Gene Reference Assay (4465807, Hs00000172_rf), following the manufacturer’s instructions (Life Technologies; http://www.lifetechnologies.com) in the Step One device. The percentage of *BRAF*^V600E^ mutations was determined from the dCT (CT reference − CT mutant) using Mutation Detector^™^ software [13]. To avoid PCR inhibition by melanin pigments, bovine serum albumin (BSA) from New England BioLabs (Hitchin, UK) was added to the PCR reactions in moderately and heavily pigmented lesions (16 cases) at a final concentration of 0.1 μg/μl [14].

### Next-generation sequencing:

BRAF NGS was performed for 11 cases (8 cases with + ve IHC/wild type CAST-PCR) and three cases that failed PCR analysis. A pooled barcoded amplicon-tagged library was generated for BRAF V600E mutation using Fluidigm^®^ Access Array TM (Fluidigm, USA) and ~ 10 pg of diluted amplicon library was taken for direct input into the emulsion polymerase chain reaction (emPCR) using Ion one touch2 (Thermo Scientific, USA) and further enriched using Ion ES station following the manufacturer’s instructions (Thermo Scientific, USA). The enriched amplicon library was sequenced using a 520-chip with an Ion S5XL semiconductor sequencer (Thermo Scientific, USA) following the manufacturer’s instructions. Targeted hotspot mutations at 599–600, as reported in the Catalogue on Somatic Mutations in Cancer (COSMIC) in the BRAF gene (covering a total of 8 amplicons), were analysed. Sequence reads were aligned to the reference genome hg19, and binary alignment map (BAM) files were generated using Ion Torrent suite version 4.0.1. The BAM files were visualized using an integrative genomics viewer (IGV) with the appropriate browser.

### Statistical analysis:

Statistical analysis was performed using SPSS version 20 (IBM). Sensitivity (PPA), specificity (NPA), positive predictive value (PPV), negative predictive value (NPV), diagnostic accuracy (overall agreement) and overall accuracy for IHC in detecting BRAF V600E in reference to CAST-PCR. (p < 0.05) were calculated. Agreement between both methods for BRAF V600E immunohistochemical staining (IHC) positivity and pattern and CAST-PCR was calculated using the ĸ agreement coefficient.

## Results

**Fig. 1:**
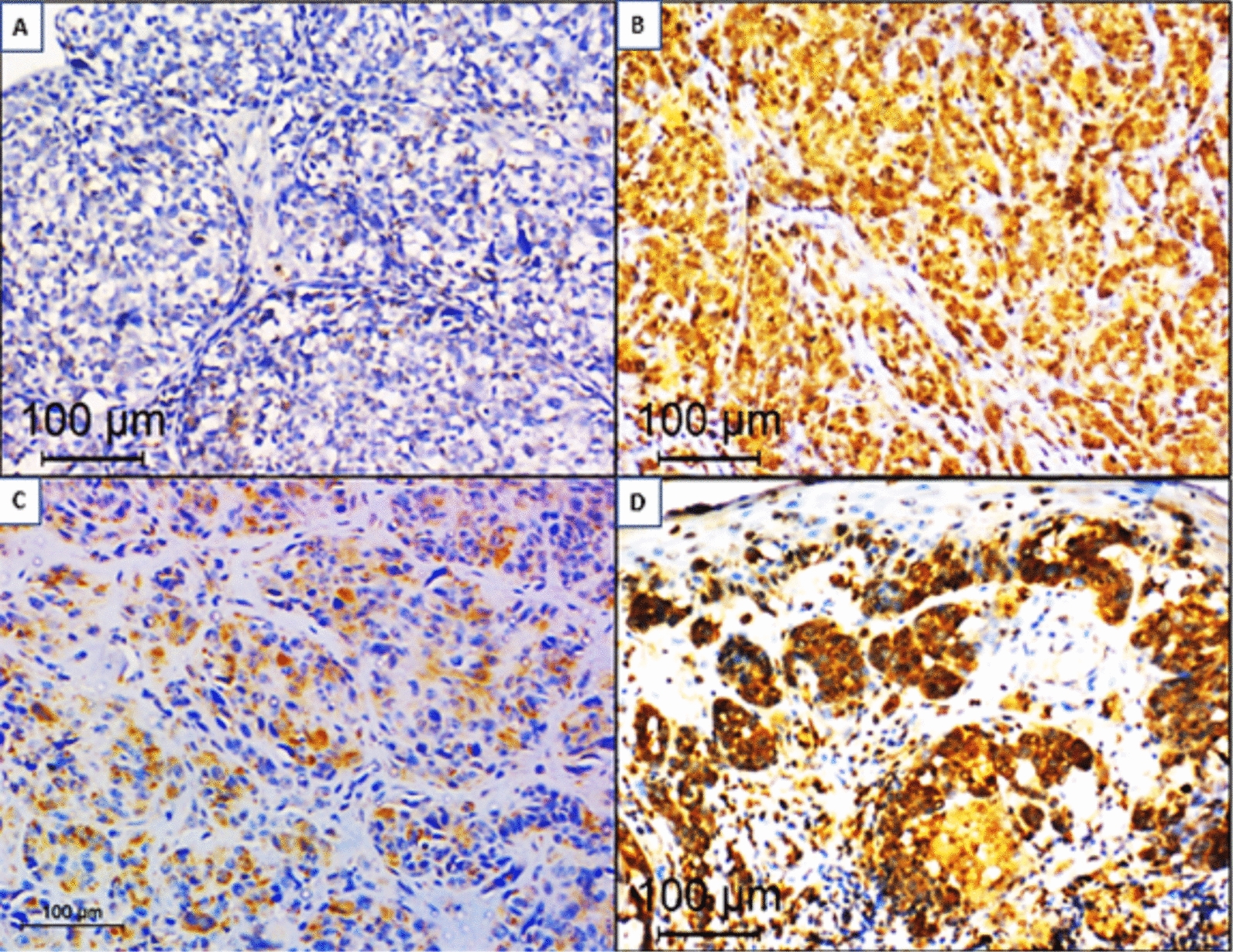
Four cases of malignant melanoma stained for BRAF^V600E^. protein (immunoperoxidase × 200) demonstrating: **A**: Negative cytoplasmic staining in tumour cells (no bleaching) **B**: Ambiguous staining in tumour cells in the form of nuclear and cytoplasmic staining (prior bleaching) **C**: Heterogenous positive cytoplasmic staining pattern (no bleaching). **D**: Homogenous positive cytoplasmic staining pattern. (prior bleaching)

The 50 studied cases were tested by IHC using a monoclonal antibody (RM-08) specific to the BRAF^V600E^ protein. Before IHC staining, melanin bleaching was performed on 12 nevi and 17 melanoma cases with pigmentation scores of 2 + and 3 + (Fig. 1). The IHC results are summarized in Table 1.

**Table 1:** IHC results of *BRAF*^*V600E*^ mutation status in all 50 studied cases of melanocytic tumours

IHC staining patterns	Number & percentage of cases		Total
	Nevi	Melanoma	
Positive	2(9.5)	17(58.6)	19 (38)
Homogenous	2	7	9
Heterogenous	0	10	10
Negative	19 (90.5)	10 (34.5)	29 (58)
Ambiguous	0	2 (6.9)	2(4)
Total	21(100)	29 (100)	50 (100)

**Fig. 2:**
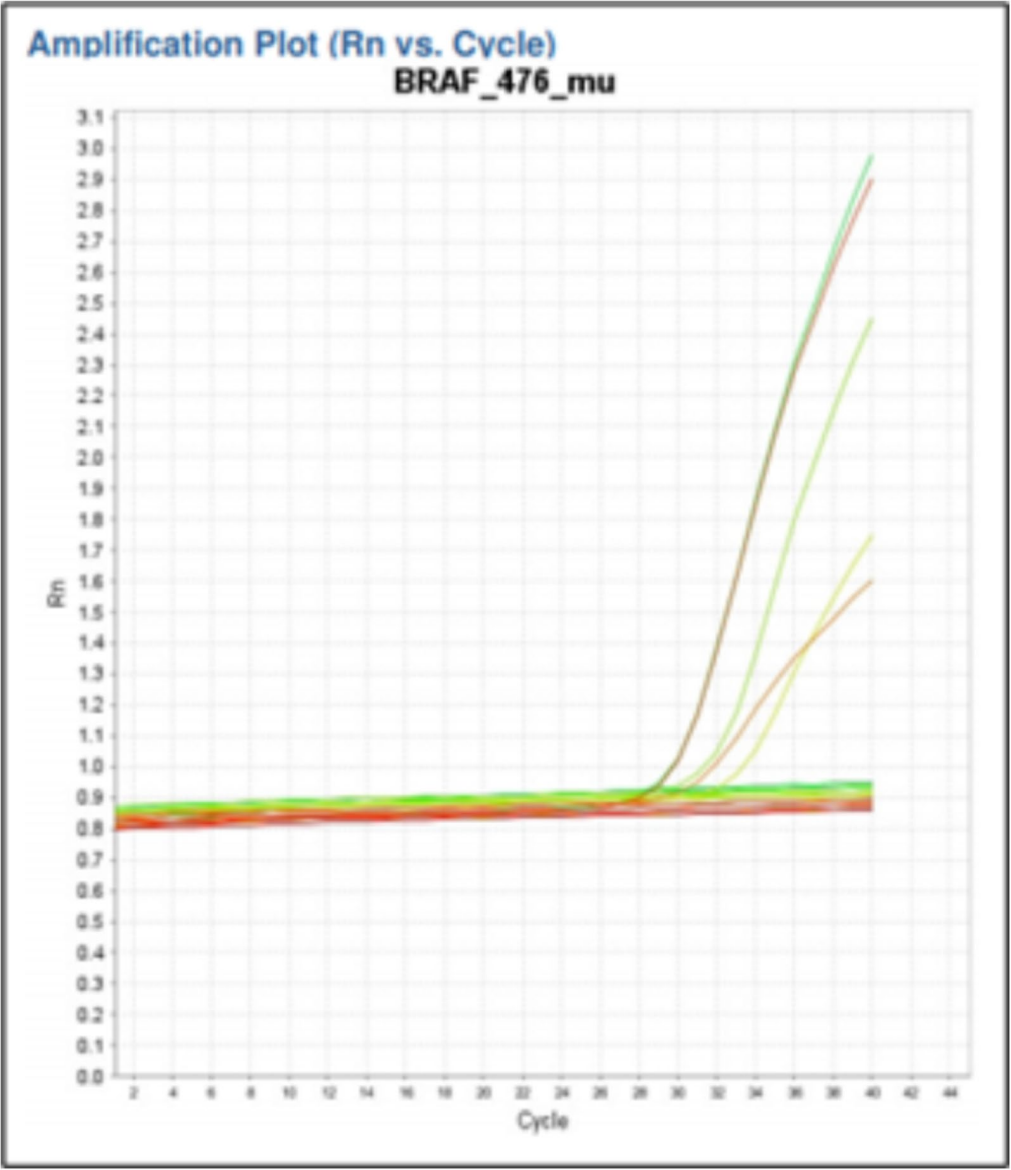
Amplification plot of the *BRAF*^V600E^ gene

Prior to *BRAF* ^V600E^ genetic analysis using CAST-PCR, BSA was applied as a melanin bleaching agent to 28 samples (12 nevi and 16 melanoma cases). *BRAF* ^V600E^ genetic analysis was successful in 44/50 cases of melanocytic neoplasms (Fig. 2).

**Fig. 3:**
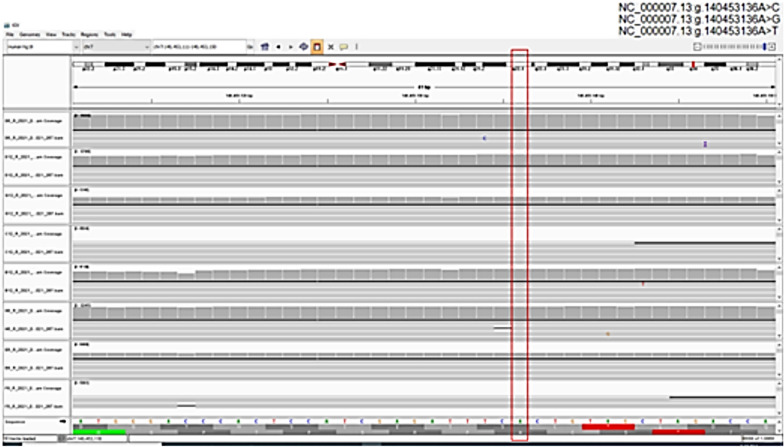
BAM files analysed for the *BRAF*^*V600E*^ mutation (chromosome 7 in Integrative genome viewer (IGV). The input files were aligned to the human hg19 reference genome

To resolve the discrepancy in eight cases with BRAF heterogonous staining patterns and *BRAF*^*Wildtype*^, (em) PCR was performed using Fluidigm^®^ for library preparation and revealed *BRAF* ^*wild type*^ in all cases (Fig. 3).

Comparison of the results of *BRAF*^*V600E*^ mutational status by IHC and CAST-PCR in the 42 studied cases are summarized in Table 2 after exclusion of ambiguous cases by IHC (2 cases) and cases that failed the PCR analysis (6 cases).

**Table 2:** Comparison of the *BRAF*^*V600E*^ mutational status by IHC and CAST-PCR in the 42 studied cases of melanocytic neoplasms

IHC results		BRAF^V600E^ CAST-PCR		Total	k-agreement p-value
		Mutant type	Wild type		
Staining reaction	Positive	8 (a) 66.67%	8 (c) 26.7%	16 (38.1%)	0.364, p = 0.16
	Negative	4 (b) 33.3%	22 (d) 73.3%	26 (61.9%)	
Staining pattern	Homogenous	8 (a) (66.67%)	0 (c)	8	K = .0.741, p = .00*
	Heterogenous/absent	4 (b) (33.3%)	30 (d)	34	

PPV (sensitivity) = a/a + b, NPA (specificity) = c/c + d, Overall percent agreement (diagnostic accuracy) = a + d/total(n)

*K* kappa agreement coefficient, *p* probability

*significant

Regardless of staining patterns, the overall agreement between both procedures was 30/42 melanocytic neoplasms (71.4%). After regrouping the cases into group 1 (homogenous staining) and group 2 (heterogeneous and absent staining), the overall agreement between both procedures was 38/42 melanocytic neoplasms (90.48%). The statistical performance of the two different evaluation IHC BRAF methods (with or without staining patterns) is summarized in Table 3.

**Table 3:** Diagnostic Accuracy of IHC Testing for Detection of the *BRAF*^*V600E*^ Mutation in the 42 studied melanocytic neoplasms in reference to CAST-PCR results

Diagnostic Accuracy	IHC BRAF ^V600^ interpretation	
	Positive vs Negative (test 1)	(Absent + Heterogeneous) vs (Homogenous) (test 2)
Sensitivity	66.7%	66.7%
Specificity	73.3%	88.2%
PPV	72.22%	72.22
NPV	71.43	100%
Diagnostic accuracy	69.9%	77.3%

*PPV* Positive predictive value, *NPA* negative predictive value

To evaluate the effects of bleaching on BRAF IHC results, mparison of concordance between IHC results with or without prior bleaching and CAST-PCR results was performed. There was no statistically significant difference in the IHC results (X2 = 3.803, P (MC) = 0.350) (Table 4).

**Table 4:** Comparison of IHC results according to bleaching with CAST-PCR results for the detection of the *BRAF* V600E mutation in the 42 studied melanocytic neoplasms using the chi-square test

IHC results in relation to CAST-PCR	Bleaching status		Total
	No bleaching	Prior bleaching	
True negative	10	13	23
True positive	3	5	8
False positive	4	4	8
False negative	3	0	3
Total	20	22	42
X^2^ P(MC)	3.803 0.350 NS		

*MC* Monte Carlo significance test, *NS* Non significant

The *BRAF* ^V600E^ mutational status was compared between nevi and melanoma cases in the current study. The *BRAF*^V600E^ mutation was detected in 6/18 nevi (33.3%) cases and 6/26 melanoma cases (23.1%) using the chi-square test, with an overall mutation rate of 27.3% (12/44).

The relationship between *BRAF* mutational status and different clinicopathological parameters was assessed in melanoma cases using the chi-square test after exclusion of cases that failed PCR analysis (3 cases). There was no statistically significant difference in *BRAF*^V600E^ PCR results among the tested parameters (Table 5).

**Table 5:** Relationship between BRAF mutational status detected by CAST-PCR and different clinicopathological parameters in 26 studied melanoma cases using the chi-square test

Clinicopathological Parameters	CAST-PCR		P-value
	*BRAF*^*V600E*^ (N = 6)	*BRAF *^*wild type*^ (N = 20)	
Gender			
Male	2 (33.3%)	9 (45%)	P(FE) = 0.674 NS
Female	4 (67.7%)	11 (55%)	
Anatomic site			
Acral	3 (50%)	13 (65%)	P_(FE)_ = 0.644 NS
Non acral	3 (50%)	7 (35%)	
Histologic type			
SSM	0	2 (10%)	p_(MC)_ = 0.710 NS
LMM	1 (16.7%)	1 (5%)	
NM	2 (33.3%)	4 (20%)	
ALM	3 (50%)	9 (45%)	
Melanoma, NOS	0	4 (20%)	
Tumour size			
T1	0	1 (5%)	P_(MC)_ = 0.445 NS
T2	1 (16.7%)	0	
T3	2 (33.3%)	8 (40%)	
T4	3 (50%)	11 (55%)	
Ulceration			
Detected	2 (33.3%)	11 (55%)	P_(FE)_ = 0.568 NS
Not detected	4 (66.7%)	9 (45%)	
In situ component			
Detected	2	12	P_(FE)_ = 0.365 NS
Not detected	4	8	
Lymphovascular invasion			
Detected	2 (33.3%)	7 (35%)	P_(FE)_ = 1.00 NS
Not detected	4 (66.7%)	13 (65%)	
Neurotropism			
Detected	0	2 (10%)	P_(FE)_ = 1.00 NS
Not detected	6	18 (90%)	
Tumour-infiltrating lymphocytes			
No TILs	1 (16.7%)	2 (10%)	p_(MC)_ = 0.335 NS
TILs identified, nonbrisk	4 (66.7%)	18 (90%)	
TILs identified, brisk	1 (16.7%)	0	
Regression			
Present	2 (33.3%)	4 (20%)	P_(FE)_ = 0.596
Absent	4 (66.7%)	16 (80%)	
Nodal status (assessed only in six cases)			
Involved	2 (100%)	3 (75%)	p_(FE)_ = 0.667 NS
Not involved	0	1 (25%)	

*FE* Fisher’s exact test, *MC* Monte Carlo significance test, *NS* Non significant

Nonparametric correlations between *BRAF* mutational status and the following parameters were performed: age, Breslow thickness, Clark level and mitotic rate using **the Spearman correlation** coefficient test. There were no statistically significant correlations between mutation detection by CAST-PCR and the tested parameters. (P = 0.270, 0.233, 0.749, 0.588), respectively.

## Discussion

In the present work, the use of Immunohistochemistry to detect BRAF^V600E^ is effective and cheap method especially when staining pattern was used with good overall agreement between both IHC and CAST-PCR results.

In current study, the overall agreement (concordance) between *BRAF*^*V600E*^ mutation detection using CAST-PCR and IHC staining reaction interpretation was 71.4%. ĸ agreement coefficient was 0.364 and statistically significant (K = 0.0.364, p = 0.016). The sensitivity and specificity were 66.7% and 73.3%, respectively. In multiple published series, concordance between IHC and CAST-PCR ranged from 88 to 97% and reported IHC sensitivity up to 97% and specificity up to 100% in detecting *BRAF*^*V600E*^ mutation.

The discrepancy between the literature data and the current work could be explained by preanalytical and analytical factors and post analytical IHC. Preanalytic include different specimen sizes (incisional biopsies vs excision), delay in the time of fixation (cold ischaemia), fixation duration and tissue-fixative ratio.

In terms of the analytical phase, bleaching before IHC may have an effect on the antigen retrieval process and the use of different antibody clones (RM-8). **Zhang et al.** when trying prior bleaching before VE-1 IHC, detected false negative staining [15]. The other studies utilized VE1 provided by Roche^®^ or Spring Bioscience^®^ without prior bleaching [10, 16, 17]. Monoclonal anti-BRAF antibodies other than the VE1 clone were reported to have lower specificity and sensitivity in *BRAF*^*V600E*^ mutation detection [18].

In terms of the postanalytic phase, a lack of training on interpretation may provide a source of interobserver variability [9, 17]. The most widely utilized method in clinical practice was proposed by Capper et al. [10, 17] and was adopted in the present study. The reproducibility of this method was argued, especially in heavily pigmented melanomas, and more stringent criteria were proposed by Fisher and colleagues [9]. Yancovitz and colleagues [12] reported that intratumoral heterogeneity in BRAF expression caused a marked discrepancy between* BRAF*^*V600E*^ molecular testing methods. Recently, a meta-analysis by Ito et al. [19] included multiple studies highlighted BRAF inter-and intratumoral heterogeneity as a possible culprit in the discrepancy between IHC results and PCR, which was resolved by sequencing.

The studied cases were reanalyzed according to the staining pattern proposed by Yancovitz after regrouping the cases into group 1 (homogenous staining) and group 2 (heterogeneous and absent staining). The specificity increased from 73.3 to 88.2%. NPA increased from 73.3 to 100%. The diagnostic accuracy increased from 71.4 to 90.48%. The overall accuracy increased from 69.9 to 77.3%. The sensitivity, positive percent agreement and positive predictive value remained unchanged. The K-agreement coefficient between both testing methods (IHC staining pattern vs CAST-PCR) increased from 0.364 (fair agreement) to 0.741 (good agreement) and was statistically significant.

Eight cases with discrepant results (^*IHC*+*ve/BRAF wild−type*^) and three cases that failed PCR analysis underwent next-generation sequencing (NGS). It revealed *BRAF *^*wild−type*^ in all eleven cases, confirming the CAST-PCR results in eight BRAF wild-type cases and detecting mutational status in three cases that failed PCR analysis. NGS can be used in *BRAF*.^*V600E*^ mutation detection with better results when compared to conventional sequencing techniques and can pick up mutations that were missed by PCR, as reported by Reiman [20]. Nevertheless, the use of PCR-based tests was reported to be a more rapid, sensitive, specific and cost-effective method for detecting BRAF mutations [21–24].

The overall mutation rate in the studied cases is less than that in the published series. Anatomic distribution and histologic subtype are strong predictors of *BRAF*^*V600E*^ mutational status in different population groups. Acral location and ALM are reported to show a lower incidence of *BRAF*^*V600E*^ mutation [25–27]. *BRAF*^*V600E*^ mutation was reported to be more frequent in nevi of compound histology with a more nesting pattern and the intraepithelial ascent of cells in comparison to dermal nevi [28]. The present work included 13 dermal nevi cases (61.9%).

The ***clinical and pathological differences*** in melanoma cases with and without the *BRAF* ^*V600E*^ mutation were assessed. *BRAF*^*V600E*^ was found to show no statistically significant correlation or association. These findings were following published data, as *BRAF *^*V600E*^ mutational status did not show any association with established prognostic factors [29]. However, its predictive value has been established in clinical practice [30].

## Conclusions

Considering the BRAF heterogeneity of melanoma, a single biopsy may not be sufficient to uncover the entire BRAF status of a patient, and multiple samples from different sites may be preferable. The results obtained in this study indicate that the IHC method cannot replace molecular methods for the detection of the BRAF^*V600E*^ mutation. There is no consensus on BRAF IHC staining interpretation criteria among different study groups, which in turn questions the methodology that should be adopted for staining interpretation.

“To our knowledge, this is the first study in the MENA region that compares BRAFV600E in Nevi and melanoma cases using different detection methods. The present work is the first to highlight the technical difficulties in BRAFV600E immunohistochemistry interpretation in terms of the assessment of heavily pigmented cases and the use of bleaching as cheap and effective method to remove melanin prior to staining. The study was conducted on 50 specimens only due to unavailability of paraffin blocks of melanocytic neoplasms as melanoma is uncommon disease in Egypt. Few statistical data are available on disease burden. Even more no public awareness on removal of melanocytic nevi and how to identify abnormal “moles” to seek medical advice. The research is self funded by the research team.

## Data Availability

Raw data sharing does not apply to Immunohistochemistry as no datasets were generated. PCR raw data and NGS are available upon request from the corresponding author.
